# Transcriptome Profiling of the Theca Interna in Transition from Small to Large Antral Ovarian Follicles

**DOI:** 10.1371/journal.pone.0097489

**Published:** 2014-05-15

**Authors:** Nicholas Hatzirodos, Katja Hummitzsch, Helen F. Irving-Rodgers, Raymond J. Rodgers

**Affiliations:** Research Centre for Reproductive Health, Discipline of Obstetrics and Gynaecology, School of Paediatrics and Reproductive Health, Robinson Research Institute, University of Adelaide, Adelaide, Australia; University of Nevada School of Medicine, United States of America

## Abstract

The theca interna layer of the ovarian follicle forms during the antral stage of follicle development and lies adjacent to and directly outside the follicular basal lamina. It supplies androgens and communicates with the granulosa cells and the oocyte by extracellular signaling. To better understand developmental changes in the theca interna, we undertook transcriptome profiling of the theca interna from small (3–5 mm, n = 10) and large (9–12 mm, n = 5) healthy antral bovine follicles, representing a calculated >7-fold increase in the amount of thecal tissue. Principal Component Analysis and hierarchical classification of the signal intensity plots for the arrays showed no clustering of the theca interna samples into groups depending on follicle size or subcategories of small follicles. From the over 23,000 probe sets analysed, only 76 were differentially expressed between large and small healthy follicles. Some of the differentially expressed genes were associated with processes such as myoblast differentiation, protein ubiquitination, nitric oxide and transforming growth factor β signaling. The most significant pathway affected from our analyses was found to be Wnt signaling, which was suppressed in large follicles via down-regulation of *WNT2B* and up-regulation of the inhibitor *FRZB*. These changes in the transcriptional profile could have been due to changes in cellular function or alternatively since the theca interna is composed of a number of different cell types it could have been due to any systematic change in the volume density of any particular cell type. However, our study suggests that the transcriptional profile of the theca interna is relatively stable during antral follicle development unlike that of granulosa cells observed previously. Thus both the cellular composition and cellular behavior of the theca interna and its contribution to follicular development appear to be relatively constant throughout the follicle growth phase examined.

## Introduction

The mammalian ovary produces oocytes for fertilization and the hormones estradiol and progesterone. Oocytes mature in ovarian follicles surrounded by pregranulosa cells at the primordial follicle stage and by granulosa cells which start replicating at the primary follicle stage. Both cell types are surrounded and separated from the ovarian stroma by the follicular basal lamina. At about the time when a fluid-filled antrum forms in the middle of the follicle, a specialized thecal layer differentiates within the stroma adjacent to the follicular basal lamina. The major functions of the thecal layer are to produce androgens, which are used by granulosa cells for estradiol synthesis, and to supply nutrients and structural support for the growing follicle. This layer can be divided into the theca interna, which contains the fibroblasts, endothelial cells, immune cells and androgen-producing cells, and the theca externa, which contains fibroblast-like cells and larger vasculature elements.

The early stages of thecal cell recruitment from ovarian stromal cells and differentiation into functional thecal cells are considered to be controlled by paracrine factors secreted by granulosa cells and oocytes (reviewed in [1], [2]). After the primary follicle stage, it has been suggested that stem cells located in the stroma [3]–[5] are induced to proliferate by stem cell factor [6] and insulin-like growth factor-1 (IGF-1) [7], which are both secreted by the granulosa cells, and oocyte-secreted factors such as growth differentiation factor 9 [3], [8]–[10]. The steroidogenic cells of the theca interna express luteinizing hormone receptor (LHCGR) and the enzymes necessary for the production of androgens including: cholesterol side-chain cleavage enzyme (CYP11A1), 3β-hydroxysteroid dehydrogenase (HSD3B) and 17α-hydroxylase (CYP17A1) [11]. They also express insulin-like factor 3 (INSL3) [12]. The proliferation, differentiation and steroidogenesis of the steroidogenic cells in the theca interna is mainly under the external control of luteinizing hormone (LH) which is secreted by the anterior pituitary [13], [14]. Recently it has been shown in thecal cell cultures, that INSL3 might play a role in maintaining androgen synthesis, while bone-morphogenetic proteins (BMPs) can act as suppressors of androgen production by inhibiting INSL3 action [15]. Activin can also suppress androgen synthesis [16] and inhibins can antagonize BMP [15] and activin actions [16]. The steroidogenic cells continue to produce androgen continuously until ovulation providing sufficient precursors for the increasing production of estradiol by the granulosa cells.

The development of a healthy follicle with the opportunity to ovulate depends on a sufficient supply of hormones, e.g. gonadotropins and growth factors, and oxygen and metabolites via the blood stream. Early follicle stages depend upon the vascular system of the ovarian stroma for their supply, whereas antral follicles have an autonomous capillary network provided by the theca interna and externa [17], [18]. The establishment of the thecal vascular network is induced and regulated by granulosa cell-secreted factors such as vascular endothelial growth factor (VEGF), basic fibroblast growth factor (FGF2), epidermal growth factor, IGF-1 and transforming growth factor β (TGFβ) (reviewed in [18]). The mRNA for VEGF and its receptor, FGF2 receptor and IGF-1 receptor have been shown to be expressed in the bovine theca interna and increase with further development of the antral follicle [19], [20]. Furthermore, receptors for angiotensin II, a vasoconstrictor, are expressed in the theca interna and even stronger in the theca externa of the bovine ovary [21], [22].

In small antral follicles (<5 mm), two types of follicles have been classified based upon the appearance of the follicular basal lamina in electron microscopic studies, in particular, follicles with an aligned or a loopy basal lamina [23]. Interestingly, antral follicles larger than 5 mm show only the aligned basal lamina type. The morphology of the follicular basal lamina at this stage has been linked to oocyte competence [24]. Additionally the shape of the basally-situated granulosa cells is related to the basal lamina phenotype with rounded cells present in follicles with an aligned basal lamina and columnar cells in follicles with a loopy basal lamina [23].

Microarray analysis of bovine preovulatory follicles before and after LH surge showed that only 2% of the 11,000 genes expressed in preovulatory follicles were differentially expressed in cells of the theca interna after the LH surge [25]. Genes involved in steroidogenesis (*CYP17A1, CYP11A1, HSD3B1, STAR*), gonadotropin receptors (*LHCGR*) and cell proliferation/cycle (*CCND2, PCNA*) were down regulated, whereas pentraxin 3 (*PTX3*) and TIMP metallopeptidase inhibitor 1 (*TIMP1*) were up regulated after LH surge [25]. Furthermore, these cells appeared to be less affected by the LH surge than the corresponding granulosa cells [25].

To further investigate the changes which occur in the theca interna during antral follicle development but prior to the effect of LH, we collected cells from the theca interna from small healthy follicles of both follicular basal lamina types (3–5 mm) and large (9–12 mm) healthy bovine follicles and identified differentially expressed genes by microarray analyses.

## Materials and Methods

### Bovine ovarian follicle selection

Pairs of ovaries were collected from non-pregnant cycling *Bos taurus* heifers at an abattoir (T&R Pastoral, Murray Bridge, SA, Australia). Follicles in two size ranges of external diameter (3–5 mm and 9–12 mm) as measured by callipers corresponding approximately to the stages of pre- and post-deviation were dissected for classification and analysis. Granulosa cells were scraped from each follicle with a Pasteur pipette tip, previously blunted by heating with a Bunsen burner, and the granulosa cells were removed. The theca interna was then dissected from the follicle wall under a Zeiss Stemi D4 stereomicroscope (Zeiss Pty Ltd., North Ryde, NSW, Australia) in ice-cold Hank's balanced-salt solution with Mg^2+^ and Ca^2+^ (Sigma-Aldrich, Castle Hill, NSW, Australia) and stored at −80°C prior to RNA extraction. An excised portion of the follicle wall (2×2×2 mm) was taken prior to granulosa and thecal cell removal and fixed in 2.5% glutaraldehyde in 0.1 M phosphate buffer for histological assessment. Follicles were classified as healthy or atretic based upon the morphology of the membrana granulosa and the presence or absence of apoptotic cells, as previously described [23], [26]. Healthy follicles were chosen for further analysis and the small follicles were classified into rounded or columnar as determined by the shape of the granulosa cells forming the layer closest to the follicular basal lamina [23].

### RNA preparation and microarray analyses

RNA was extracted from thecal cells by the Trizol method (Life Technologies, Mt Waverley, VIC, Australia). Briefly, each thecal sample was homogenized in 1 ml of Trizol with 1.4 mm ceramic beads in a Precellys 24 Bead Mill Homogenizer (Omni International, Kennesaw, Georgia, USA) with two 10 s cycles of 6,000 rpm each. The samples were then extracted with 200 µl of chloroform and the aqueous phase was purified through a Qiagen RNEasy mini prep column (Qiagen, Hilden, Germany) according to the manufacturer's instructions. Five µg of RNA was treated to remove genomic DNA contamination with 2 units of DNAse 1 (Ambion/Life Technologies) prior to labeling for microarray analysis. All RNA samples were found to have a RNA integrity number ≥8 when assessed by microfluidic analysis on a 2000 BioAnalyzer (Agilent, Santa Clara, CA, USA).

DNAse-treated RNA (100 ng) was labeled using the 3′IVT Express labeling kit (Affymetrix, Santa Clara, CA, USA). In brief, the RNA was reverse transcribed using a T7 oligo dT primer followed by second strand synthesis. *In vitro* transcription reactions were performed in batches to generate biotinylated cRNA targets, which were subsequently chemically fragmented at 95°C for 35 min. Ten µg of the fragmented, biotinylated cRNA was hybridized at 45°C for 16 h to Affymetrix GeneChip Bovine Genome Arrays, which contain 24,027 probe sets representing over 23,000 transcripts and variants, including 19,000 UniGene clusters. The arrays were then washed and stained with streptavidin-phycoerythrin (final concentration 10 µg/ml). Signal amplification was achieved by using a biotinylated anti-streptavidin antibody. The array was then scanned according to the manufacturer's instructions (Affymetrix GeneChip Expression Analysis Technical Manual). The arrays were inspected for defects or artefacts. The array data was converted to CEL file format for analysis.

### Microarray data analysis

The quality control for the cDNA labeling was determined by the use of internal array controls. The array data were subjected to Robust Multi-Array Average summarization [27] and quantile normalization [28] which was considered to be statistically appropriate treatment for normally distributed data for arrays of this size (greater than 20,000 probe sets). Probe sets were filtered such that only those with a log_2_ signal intensity of >3.0 for ≥50% of the arrays of one follicle type were considered to be above the detection threshold. The fold change determination and statistical analysis of the data were performed as detailed previously in [29]. The microarray CEL files, normalized data and experimental information have been deposited in the Gene Expression Omnibus [30], and are available by the accession number GSE49505.

Function, pathway, network and upstream regulator analysis were conducted in IPA and GOEAST similarly as described in previous studies [29], [31].

### Measurement of gene expression by quantitative RT-PCR

Total RNA (200 ng) from the theca interna of small and large healthy follicles (n = 7 and n = 4) was extracted and used to synthesize cDNA similarly as detailed previously [32]. Real time RT-PCR assays were designed against nine genes using web based software and quantitative RT-PCR was performed as further described in [32]. The sequence information of the primers used for quantitative RT-PCR is shown in Table 1.

**Table 1:** Primer sequences used for qRT-PCR.

Gene Name	Gene Symbol	GenBank Accession No.	Forward Primer (5′- 3′)	Reverse Primer (5′- 3′)	Product Size (bp)
Glyceraldehyde 3-phosphate dehydrogenase	*GAPDH*	XR_027767	ACCACTTTGGCATCGTGGAG	GGGCCATCCACAGTCTTCTG	76
Peptidylprolyl isomerase A (cyclophilin A)	*PPIA*	NM_178320.2	CTGGCATCTTGTCCATGGCAAA	CCACAGTCAGCAATGGTGATCTTC	202
Frizzled-related protein	*FRZB*	NM_174059	GTGAGCCCGTTCGCATTC	GGTTGGGCATCTTAGTCATGTTC	63
Insulin growth factor binding protein 3	*IGFBP3*	NM_174556	CGCCTGCGCCCTTACC	TTCTTCCGACTCACTGCCATT	57
Retinoic acid receptor responder (tazarotene induced) 1	*RARRES1*	NM_001075430	AAGCCCCTTGAATGCAGTCA	TGGGTCTCAGAGATGGAGCAA	65
Claudin 11	*CLDN11*	NM_001035055	TGGGTCTGCCGGCTATTCT	GGCCCATTCGGATGCA	57
Aldehyde oxidase 1	*AOX1*	NM_176668	CTGGGAGAGTCTGGGATATTCCT	CGTGCTGCCCTTATTGCAT	71
Latent TGFβ binding protein 1	*LTBP1*	NM_001103091	GATTTGGGCCAGATCCTACCT	CGGTAACACGGCCCTTTCT	79
Wingless-type MMTV integration site family, member 2B	*WNT2B*	NM_001099363	CGGACTGACCTGGTCTACTTTG	AGGGAACCTGCAGCCTTGT	67
Cyclin E2	*CCNE2*	NM_001015665	CCTCATTATTCATTGCTTCCAAAC	TTCACTGCAAGCACCATCAG	89
Centromere protein F, 350/400 kDa (mitosin)	*CENPF*	XM_002694283	CGACATCCCAACCGGAAAG	TTGGAGGTCTCGGTGAGATTTT	141

## Results and Discussion

### Statistical analyses of differentially expressed genes

Theca interna from two groups of healthy follicles, small (n = 10) and large (n = 5), were histologically classified as described in the methods and examined by microarray analysis of gene expression. The small healthy follicles were sub-classified into two groups possessing either columnar (n = 5) or rounded (n = 5) basally-situated granulosa cells. The original analysis across the three groups by one-way ANOVA did not indicate any gene differences with a minimum two-fold change and False Discovery Rate (FDR) of *P*<0.05 between the two healthy sub-groups, therefore these were treated as a single group for further analyses.

**Figure 1:**
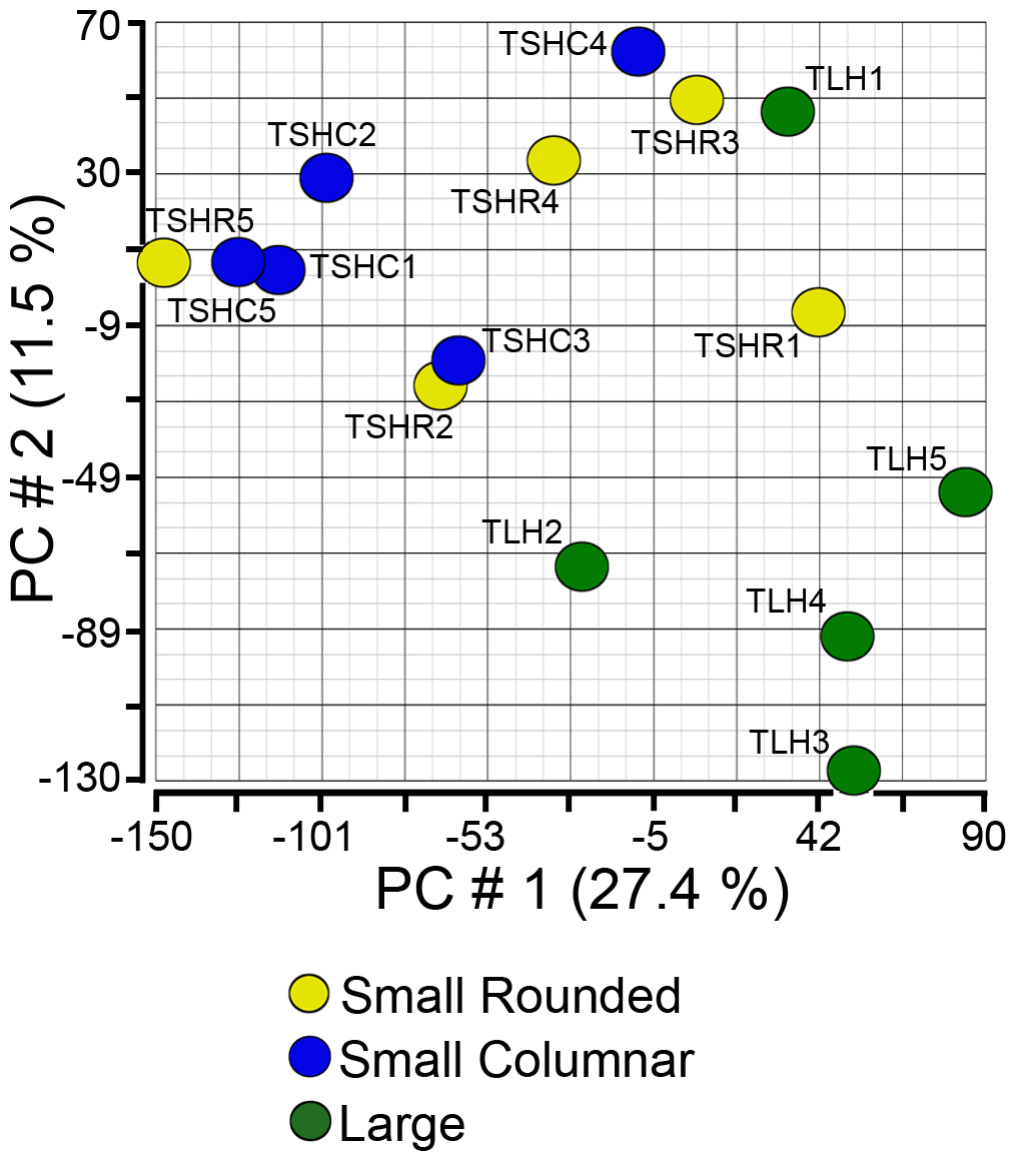
Unsupervised PCA of arrays for thecal cells from small and large healthy follicles. The graph is a scatter plot of the values for the first (X) and second (Y) principal components based on the correlation matrix of the total normalized array intensity data. Abbreviations are thecal small healthy rounded (TSHR), thecal small healthy columnar (TSHC) and thecal large healthy (TLH).

Principal Component Analysis (PCA) mapped the overall differences in gene expression between the individual arrays as shown in Fig. 1. There was some degree of relatedness based on follicle size detected by this analysis and by hierarchical clustering (Fig. S1), however, the overall differences were not as distinct when compared with granulosa cells in a similar study in our laboratory [31]. This suggests that the theca has a relatively stable transcriptional profile during antral follicle development up to the period where follicle growth becomes largely under the control of LH. One thecal sample, TLH4, was found to have relatively high expression levels of granulosa- specific genes such as *CYP19A1*, *FSHR*, *INHBA* and *FST*, and was therefore considered to be contaminated by granulosa cells and excluded from further analyses.

A total of 76 probe sets (out of 15,530 detected), representing 57 genes (Table S1), 53 of which were up regulated and 4 down regulated (Table 2), were determined to be differentially expressed between the large and small healthy theca layers (≥2 fold change, FDR *P*<0.05) by ANOVA analysis in Partek. This data set was considerably smaller than the statistically equivalent group generated for granulosa cells for the comparison of large versus small healthy follicles where more than 10% of the probe sets (n = 2714) were differentially regulated [31]. This further supports the assumption that the theca interna is quite stable and does not substantially alter overall gene expression with increasing follicle size to this stage of maturation. The most highly up regulated gene was found to be *CLDN11* (8 fold), a known tight junction marker of the blood-brain and blood-testis barriers [33], [34]. The n = 76 data set was uploaded for pathway and network analysis into Ingenuity Pathway Analysis (IPA) and into Gene Ontology Enrichment Analysis Toolkit (GOEAST) software [35].

**Table 2:** Number of probe sets and genes differentially expressed in large healthy follicles with respect to small follicles.

Fold-Change	Probe Sets			Genes		
	Up-regulated	Down-regulated	Total	Up-regulated	Down-regulated	Total
>2	71	7	76	53	4	57
>3	19	1	20	13	1	14
>4	5	0	5	3	0	3

Statistical difference with *P*<0.05 was determined by ANOVA using the step up Benjamini-Hochberg False Discovery Rate method for multiple corrections in Partek Genomics Suite Software.

### Functional and pathway analyses of differentially expressed genes

**Figure 2:**
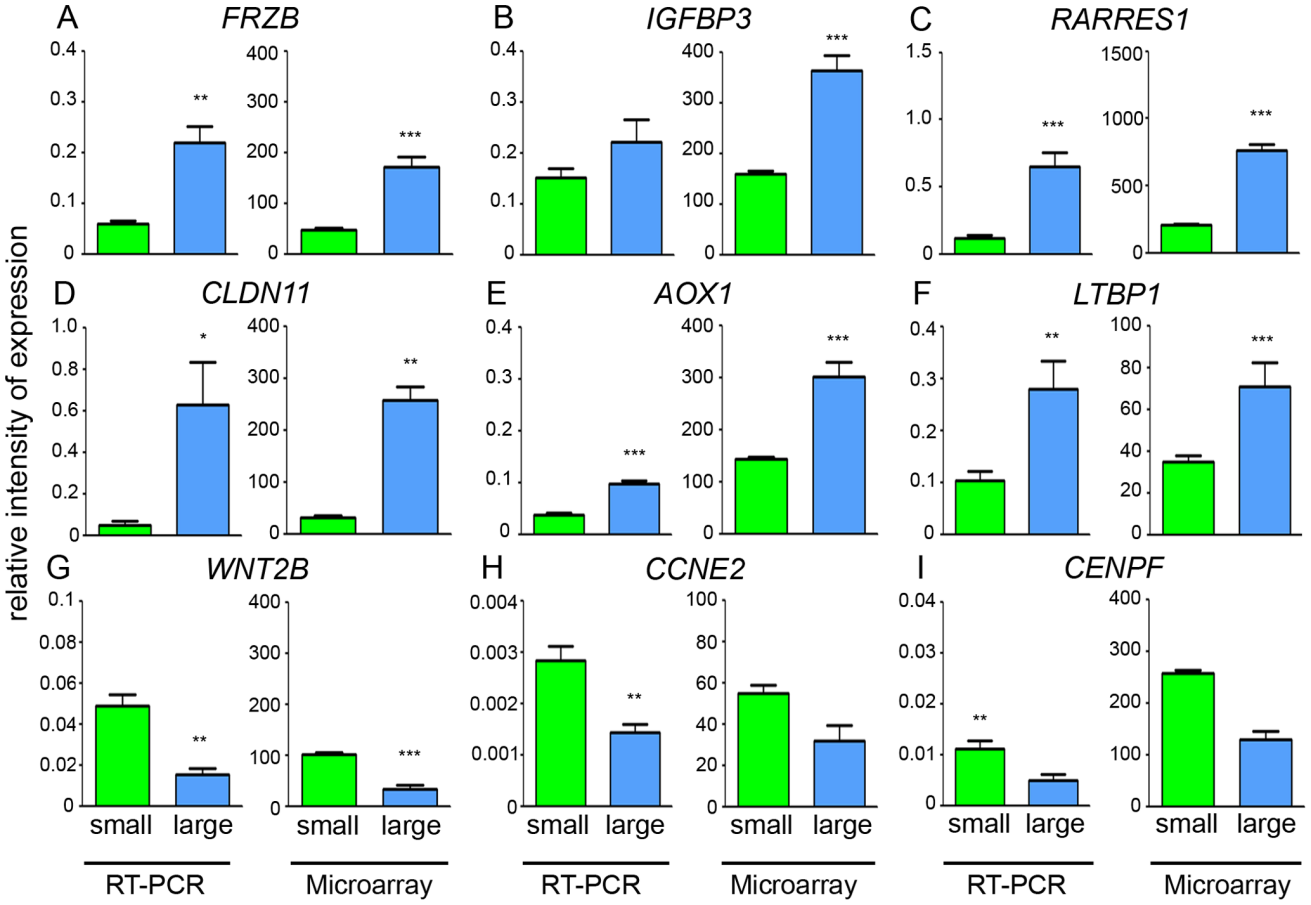
Measurement of gene expression by qRT-PCR. The data are shown as the mean ± SEM (n = 7 for small follicle group, n = 4 for large follicle group). qRT-PCR values were determined from the mean of the ratio of 2^−ΔCt^ of the target genes to cyclophilin A (*PPIA*) and glyceraldehyde phosphate dehydrogenase (GAPDH), and the microarray values are signal intensities (normalized but not log transformed). Significantly different results for qRT-PCR were determined by Student's *t*-test. The *P* values for the microarray results are corrected for multiple testing using the FDR (*P<0.05, **P<0.01 and ***P<0.001).

The expression levels of nine genes selected to include up- and down regulated genes and genes with no change between small and large follicles were determined by qRT-PCR and the results are presented in Fig. 2. The fold-change data from the arrays and the qRT-PCR experiments (Fig. 2) were highly correlated with each other (Pearson's correlation, *R^2^* = 0.95, *P*<0.001; Fig. S2), indicating that the arrays were correctly identifying differentially expressed genes. Genes which were differentially regulated between large and small follicle theca interna and eligible for network analyses in IPA are listed in Table 3, which consisted of 47 in total, including 45 up and 2 down regulated.

**Table 3:** Genes which were differentially regulated in large with respect to small healthy follicles.

Gene Symbol	Fold Change	Gene Symbol	Fold Change	Gene Symbol	Fold Change
**Cell Cycle and DNA Replication**					
DYNLT3	2.1	TOP1	3.2		
**Cell Morphology**					
MFAP5	4.0	DES	2.4	CDC42EP3	2.0
**Cytokines, Hormones and Receptors**					
NTRK2	3.1	PTPRB	2.3	CD44	2.1
IL20RA	2.8	IGFBP3	2.3	**WNT2B**	**−3.0**
NOV	2.8				
**Extracellular Matrix and Synthesis**					
LTBP2	3.0	SMOC2	2.5	TNXB	2.2
LTBP1	2.9	COL14A1	2.2		
**Intercellular and Cell to Matrix Adhesion**					
CLDN11	8.2	EPCAM	2.3		
CDH3	2.5	CCDC80	2.1		
**Proteolysis or Inhibition**					
ADAMTSL4	2.2	ANPEP	2.1		
USP7	2.1	EPHX1	2.0		
**Transcription Regulation**					
NRIP3	5.6	GAS	2.9	KLF6	2.0
FBXO32	3.6	ZNF618	2.1		
**Transport**					
RTP4	2.3				
**Other Enzymes**					
LEPREL1	3.0	AOX1	2.1		
P4HA3	2.4	LIPG	−2.7		
**Other Signalling**					
RSPO3	5.5	GBP1	2.7	HSP90AA1	2.2
RARRES1	3.7	CAV1	2.4	HSPB8	2.1
FRZB	3.6	PLN	2.3		
Other					
SCUBE2	2.9	FAM114A1	2.6	CRYAB	2.1
WDFY4	2.6	PLXDC2	2.2		
**Non IPA-annotated genes**					
LOC535166	3.6	TNC	2.3	DCLK1	2.1
RGS2	3.4	CEBPB	2.3	**FAM122B**	**−2.3**
CHD2	2.4	BST2	2.2	**LOC512149///LOC512150**	**−2.4**

Genes were ≥2 fold different with *P*<0.05 between large and small healthy follicles. *P* value determined by Benjamini-Hochberg post-hoc test for multiple corrections following one way ANOVA. Genes are listed in descending order within each functional category. Genes which are down regulated are listed in bold.

**Figure 3:**
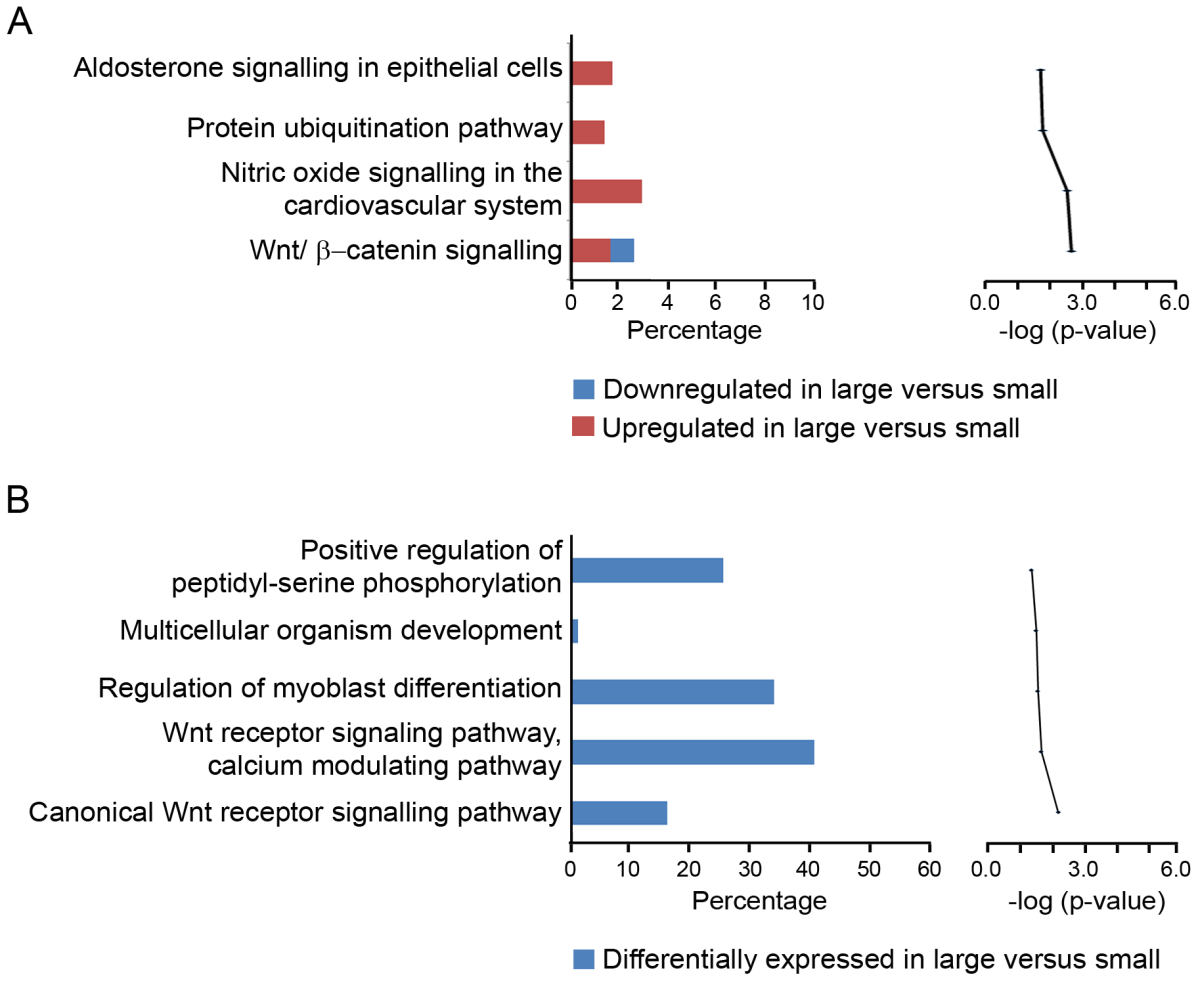
Top canonical pathways mapped in IPA (A) and GO terms (B) classified under biological process. In (A) the bar chart on the left represents the percentage of genes from the data set that map to each canonical pathway showing those which are up regulated (in red) and down regulated (in blue) in theca of large with respect to small healthy follicles. The line chart on the right ranks these pathways derived for the same data set, from the highest to lowest degree of association based on the value of a right-tailed Fisher's exact *t* test. In (B) the bar chart on the left represents the percentage of genes from the data set that map to each GO term showing those which are differentially regulated (in blue) in theca of large with respect to small healthy follicles. The line chart on the right ranks these pathways derived for the same data set, from the highest to lowest degree of association using the Benjamini-Yuketeli test for multiple corrections (bottom to top in graphs on right).

Analysis in IPA and GOEAST to determine canonical pathway and gene ontology (GO) term association showed that some molecules which were differentially regulated between large and small follicles map to the Wnt signaling pathway (Figs 3A and B, respectively). This involved the inhibition of *WNT2B*, which encodes a ligand which can activate the canonical pathway, and activation of *FRZB*, encoding a secreted frizzled receptor which modulates the effect of Wnt ligands by direct interaction and up-regulation of cadherin 3. There were also associations with myoblast differentiation, protein ubiquitination and nitric oxide signaling.

**Figure 4:**
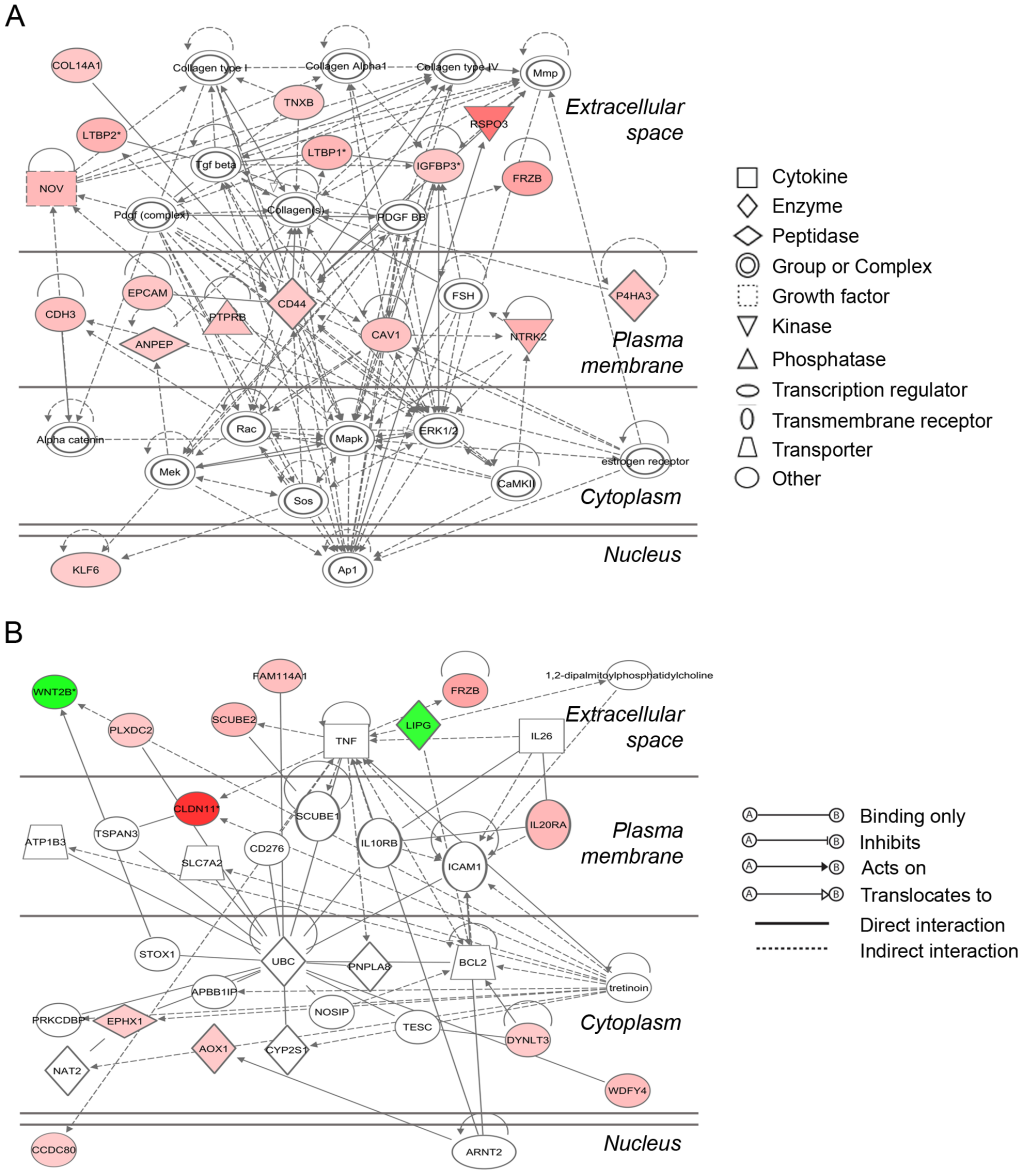
The two most significant gene networks mapped in IPA. The networks were generated in IPA using triangle connectivity based on focus genes (those present in our data set) and built up according to the number of interactions between a single prospective gene and others in the existing network, and the number of interactions the prospective genes have outside this network with other genes as determined by IPA [65]. Network A (score = 39), shows interactions between *LTBP1*, *LTBP2*, *COL14A1* and *TNXB*I indicating extracellular matrix signalling and network B (score = 28), shows involvement of Wnt pathway members *WNT2B* and *FRZB*. Interactions between molecules, and the degree and direction of regulation are indicated with up- (red) or down-regulation (green) and increasing color intensity with degree of fold change.

The two top networks generated in IPA from our differentially expressed genes are shown in Fig. 4. Fig. 4A indicates an emphasis on TGFβ signaling via *LTBP1* and *LTBP2*, and extracellular matrix synthesis via *COL14A1*, *TNXB* and *P4HA3*. There was also interaction with *IGFBP3* and particularly *CD44* which is central to this network. Fig. 4B shows associations with Wnt signaling through the molecules mentioned above and contains the only down regulated genes in the entire data set i.e. *WNT2B* and *LIPG,* or endothelial lipase.

Genes or molecules predicted to be regulated from the analysis in IPA are shown in Table 4. Those expected to be activated include *TP53, IFNG,* and the DNA hypomethylating agent decitabine, which all act to curb cell replication and growth [36]–[38]. *IL1RN*, the IL-1 receptor antagonist gene, predicted to be inhibited, plays a role in modulating the immune response [39].

**Table 4:** A list of 4 upstream regulators predicted to be activated or inhibited in IPA.

Upstream Regulator	Molecule Type	Predicted Activation State †	Bias-Corrected z-score ††	*P* Value of Overlap**	Target Molecules in Data Set
decitabine	chemical drug	Activated	2.587	3.08E-04	CAV1, CD44, CDH3, HSPB8, IGFBP3, RARRES1, RTP4
TP53	transcription regulator	Activated	2.001	7.99E-04	CAV1, CCDC80, CD44, CDC42EP3, CDH3, COL14A1, CRYAB, EPHX1, GBP1, HSP90AA1, IGFBP3, LTBP1
IFNG	cytokine	Activated	2.135	3.69E-02	CD44, GBP1, HSP90AA1, KLF6, PTPRB, RARRES1, RTP4′
IL1RN	cytokine	Inhibited	−2.000	4.49E-04	GBP1, KLF6, RARRES1, RTP4

† The predicted activation state is inferred from the bias-corrected z-score.

†† The bias-corrected z-score is computed based on the proportion of target genes present in the data set which are directionally regulated as expected according to known effects of the regulator on the target compiled from the literature.

**The *P* value of overlap measures the statistical significance of overlap using Fisher's exact *t*-test, between genes from the data set and those known to be acted upon by an upstream regulator.

### Transcriptional processes in thecal tissue during antral follicle growth

#### Wnt signaling

R-spondins, Wnt2 and the frizzled proteins all impact on Wnt signaling pathways and are active in mammalian reproductive organ and follicle development [40]–[46]. Previous studies in the ovary have mainly focused on oocytes and granulosa cells in rodents. Wnt signaling has previously been shown to be active at the preantral follicle and the preovulatory stages [41]. In this study it appears that Wnt signaling is probably down regulated in the theca interna, as the antral follicle enlarges as a consequence of lower *WNT2B* expression and higher expression of the Wnt inhibitor *FRZB*. It should be noted that the microarray did not contain probes for Wnt4 or Wnt5, previously identified as the major ligands and shown to be expressed in growing follicles. The situation is complicated by the fact that *CDH3*, which encodes P-cadherin, is also up regulated and has been shown to positively regulate Wnt signaling in hair follicles [47], though this may be a tissue-specific effect. *RSPO3* is also highly activated, but this is likely due to its known effect on the promotion of angiogenesis [48], a necessary requirement to sustain and promote follicle enlargement. We did not see differences in β-catenin levels between the follicle groups in our microarray data, suggesting that Wnt signaling in the antral follicle theca interna occurs by some non-canonical pathway.

#### Oxidative stress

Our data showed evidence of oxidative stress response in large follicle theca interna as determined by the analysis in IPA (Fig. 3A), mainly through the transcriptional activation of genes like *HSP90AA1*, *CAV1* and *CRYAB* (Table 3). Oxidative stress may be caused by production of reactive oxygen species from steroidogenesis occurring in the androgen producing cells of the theca interna [49], or additionally perhaps due to increased activity of NADPH oxidases in the vascular endothelium [50] by hypoxia-induced stimulation of angiogenic pathways to meet the needs of the growing follicle.

#### Lipid metabolism

Two genes which are concerned with lipid modification, *EPHX1*(epoxide hydrolase) and *AOX1* (aldehyde oxidase), were found to be expressed more highly in the larger follicle theca interna. Previous studies appear to link *EPHX1* activity to estrogen production [51], and together with *AOX1*, it may be involved with the removal of cytotoxic epoxide compounds formed during steroidogenesis. These higher expression levels reflect the increased steroidogenic capacity of larger follicles.

#### Extracellular matrix proliferation

A number of genes including *IGFBP3, LTBP1, NOV, LEPREL1, P4HA3* and *COL14A1* were predicted to be activated in the theca surrounding large follicles and promote fibroblast proliferation and collagen synthesis (Table 3 and Fig. 4A). Some of these, such as *IGFBP3* and *LTBP1* have been previously associated with follicle development [52]–[54]. The increase in follicle size accompanied by an enlarged and thickened thecal layer with increased collagen would explain the observed higher expression of these genes at this stage of development.

#### Cell division

There seemed to be only a minor reduction in cell division at the transcriptional level in the large follicles. The expression of two cell cycle genes *CENPF* (mitosin) and cyclin E2 was additionally examined by qRT-PCR (Fig. 2), and the results also confirmed only a slight reduction in expression of these cell cycle genes.

#### CLDN11

(Claudin-11), a tight junction protein, has been shown to be present in locations where strict homeostasis control and protection from xenobiotics is important, such as the blood-testis [34] and the blood-cerebrospinal fluid [55] barriers. There is some evidence of the influence of androgens on increased expression of claudin-11 in the Sertoli cells of the testis [56], [57]. It is possible that the gene for this protein becomes more highly expressed in response to increased production of steroids at the later antral stage of follicle development, but the functional role for an increase in expression in the theca is unknown.

#### RARRES1

(Retinoic Acid Receptor Responder 1) has been identified as a tumor-suppressor gene with probable carboxypeptidase inhibitor function [58] and it may play a role in repression of stem cell phenotype [59]. There has been only one report of expression of this gene in non-pathological ovarian function, specifically in cumulus cells [60], although we identified *RARRES1* to be down regulated by tumor necrosis factor -α in cultured granulosa cells [61]. This gene also appears to be hormonally regulated in endometrial tissue studies [62], [63], and Nguyen et al. [63] demonstrated a link between endometrium development and Wnt signaling. The significance of *RARRES1* expression in the theca interna may be due to a similar developmental function, whereby cell replication is controlled and differentiation of cells into a more mature phenotype is promoted.

## Conclusions

In this study we compared the theca interna from small and large follicles that represents a calculated >7 fold increase in the amount of thecal tissue. Observed changes in the transcriptional profile could have been due to changes in cellular function or alternatively since the theca interna is composed of a number of different cell types it could have been due to any systematic change in the volume density of any particular cell type. However, our study suggests that the transcriptional profile of the theca interna is relatively stable during antral follicle development, unlike that of granulosa cells observed previously [64]. Thus both the cellular composition and cellular behavior of the theca interna and its contribution to follicular development appear to be relatively constant throughout the follicle growth phase examined.

## Supporting Information

Figure S1 **Unsupervised hierarchical clustering across all probe sets (n = 24,182) for 15 arrays.** The analysis was performed using the Euclidian dissimilarity algorithm with the average linkage method in Partek Genomics Suite. The heatmap represents the distribution of normalized signal intensity, grouping by pattern similarity for both probe set and array. Abbreviations for identification of array samples are identical to Fig. 1.
(TIF)

Figure S2 **Scatter plot of fold changes in microarray intensity versus fold-changes in expression determined by qRT-PCR.** Values represent nine selected genes as presented in Fig. 2. The two sets of data were highly correlated with each other (Pearson's correlation, *R^2^* = 0.95, *P*<0.001).
(TIF)

Table S1 Probe sets which are up regulated in large with respect to small healthy follicles. Analysis by ANOVA in Partek, with ≥2 fold-change and *P*<0.05 (n = 76), in alphabetical order. Probe sets which do not have gene assignations are placed at the end of the list. The *P* value for multiple corrections was determined by the step up FDR method.
(PDF)
