# Supplementary figures and images for: Transcriptome Profiling of the Theca Interna in Transition from Small to Large Antral Ovarian Follicles
Source: PLoS One. 2014 May 15;9(5):e97489. doi: 10.1371/journal.pone.0097489 (PMC4022581; doi:10.1371/journal.pone.0097489)

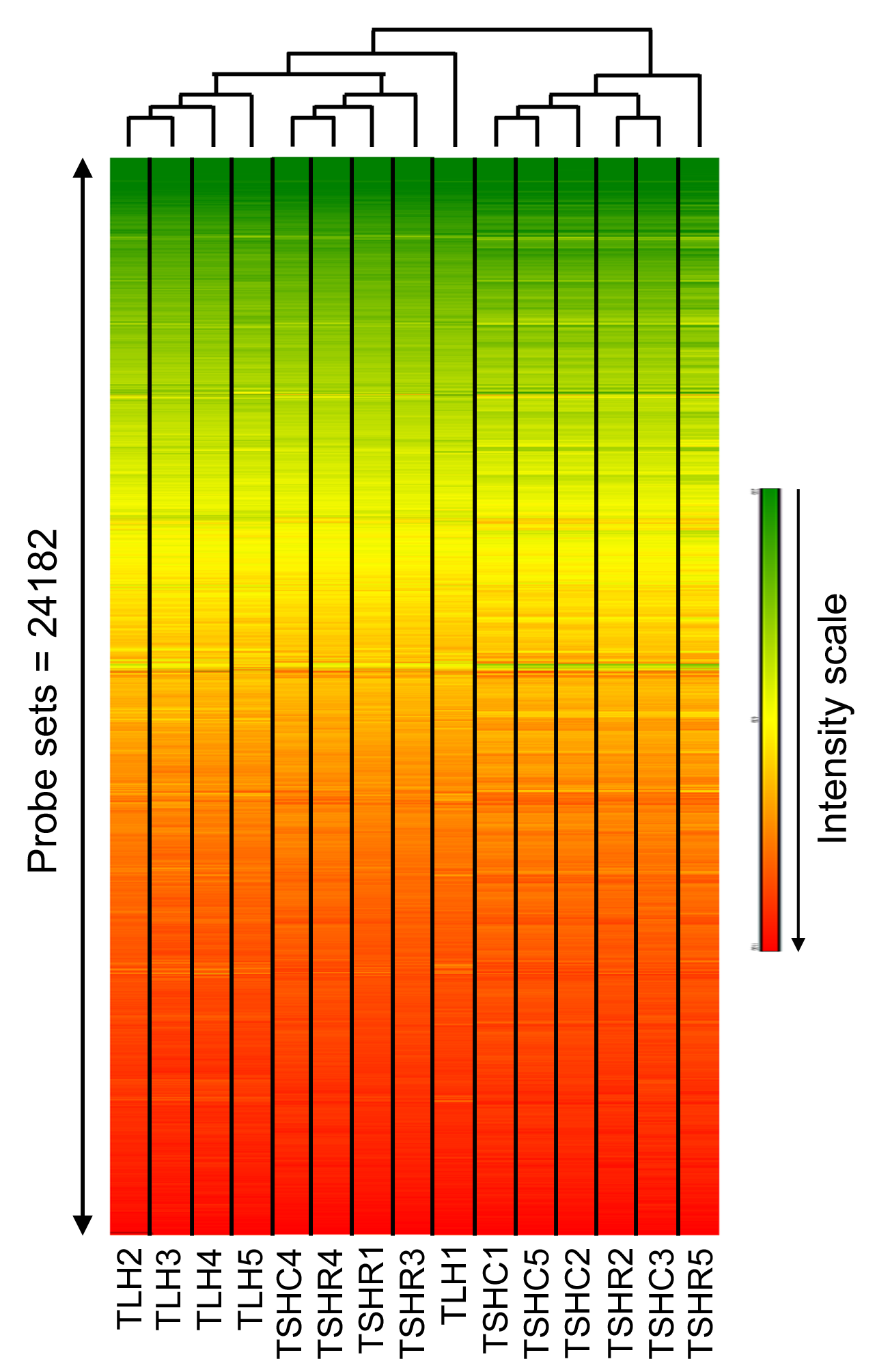

Supplement: Figure S1 — Unsupervised hierarchical clustering across all probe sets (n = 24,182) for 15 arrays. The analysis was performed using the Euclidian dissimilarity algorithm with the average linkage method in Partek Genomics Suite. The heatmap represents the distribution of normalized signal intensity, grouping by pattern similarity for both probe set and array. Abbreviations for identification of array samples are identical to Fig. 1. (TIF) [file pone.0097489.s001.tif]

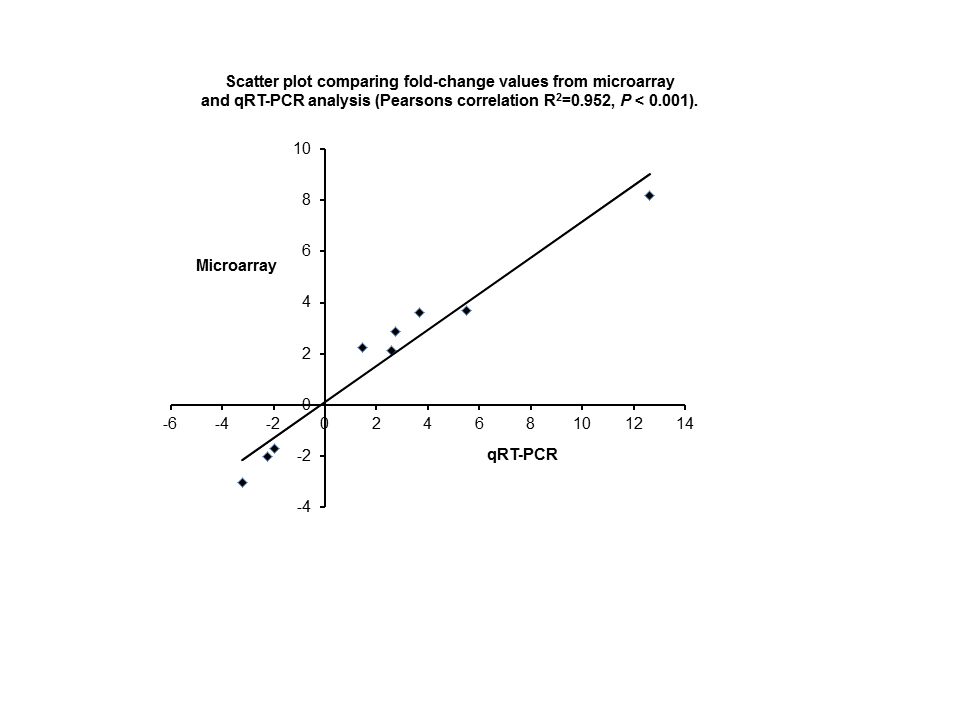

Supplement: Figure S2 — Scatter plot of fold changes in microarray intensity versus fold-changes in expression determined by qRT-PCR. Values represent nine selected genes as presented in Fig. 2. The two sets of data were highly correlated with each other (Pearson's correlation, R2 = 0.95, P<0.001). (TIF) [file pone.0097489.s002.tif]
