# Supplementary material for: Transcriptome Profiling of the Theca Interna in Transition from Small to Large Antral Ovarian Follicles
Source: PLoS One. 2014 May 15;9(5):e97489. doi: 10.1371/journal.pone.0097489 (PMC4022581; doi:10.1371/journal.pone.0097489)
Supplement: Table S1 — Probe sets which are up regulated in large with respect to small healthy follicles. Analysis by ANOVA in Partek, with ≥2 fold-change and P<0.05 (n = 76), in alphabetical order. Probe sets which do not have gene assignations are placed at the end of the list. The P value for multiple corrections was determined by the step up FDR method. (PDF) [file pone.0097489.s003.pdf]

**Probe set ID**	**Gene Symbol**	**Gene Title**	**RefSeq Transcript ID**	**Fold-Change**	***P*-value**
Bt.20236.1.S1_at	*ADAMTSL4*	ADAMTS-like 4	NM_001101061	2.162	3.11E-02
Bt.8027.1.S1_at	*ANPEP*	alanyl (membrane) aminopeptidase	NM_001075144	2.076	2.56E-02
Bt.783.1.S1_at	*AOX1*	aldehyde oxidase 1	NM_176668	2.113	3.80E-02
Bt.15788.1.S1_a_at	*BST2*	Bone marrow stromal cell antigen 2	XM_584000	2.165	4.15E-02
Bt.2230.1.S1_at	*CAV1*	caveolin 1, caveolae protein, 22kDa	NM_174004	2.415	2.32E-02
Bt.7788.1.S1_at	*CCDC80*	coiled-coil domain containing 80	NM_001098982	2.066	3.93E-02
Bt.5494.1.S1_at	*CD44*	CD44 molecule (Indian blood group)	NM_174013	2.119	2.97E-02
Bt.1560.1.S1_at	*CDC42EP3*	CDC42 effector protein (Rho GTPase binding) 3	NM_001046444	2.035	1.95E-02
Bt.3948.1.S1_at	*CDH3*	cadherin 3, type 1, P-cadherin (placental)	XM_614683	2.545	3.59E-02
Bt.22785.1.S1_at	*CEBPB*	CCAAT/enhancer binding protein (C/EBP), beta	NM_176788	2.274	3.29E-02

Bt.16252.3.A1_s_at	*CHD2*	chromodomain helicase DNA binding protein 2	NM_001102181	2.355	3.02E-02
Bt.4817.1.A1_at	*CLDN11*	claudin 11	NM_001035055	8.179	4.99E-02
Bt.4817.2.S1_at	*CLDN11*	claudin 11	NM_001035055	3.869	7.97E-03
Bt.222.1.S1_at	*CRYAB*	crystallin, alpha B	NM_174290	2.128	2.32E-02
Bt.19199.1.A1_at	*DCLK1*	Doublecortin-like kinase 1	NM_001109962	2.147	1.96E-02
Bt.6141.1.S1_at	*DES*	desmin	NM_001081575	2.372	4.15E-02
Bt.20800.1.S1_at	*DYNLT3*	dynein, light chain, Tctex-type 3	NM_001101220	2.092	4.15E-02
Bt.9569.1.S1_at	*EPCAM*	epithelial cell adhesion molecule	NM_001035290	2.282	2.00E-03
Bt.23354.1.S1_at	*EPHX1*	epoxide hydrolase 1, microsomal (xenobiotic)	NM_001034629	2.020	4.18E-02
Bt.24200.1.S1_at	*FAM122B*	family with sequence similarity 122B	NM_001083794	-2.321	1.23E-02
Bt.18639.1.A1_at	*FBXO32*	F-box protein 32	NM_001046155	3.577	2.95E-02
Bt.18639.2.S1_at	*FBXO32*	F-box protein 32	NM_001046155	3.044	3.46E-02

Bt.121.1.S1_at	*FRZB*	frizzled-related protein	NM_174059	3.630	1.49E-02
Bt.13326.1.S1_at	*GAS7*	growth arrest-specific 7	NM_001102280	2.880	5.53E-03
Bt.23179.1.S1_at	*HSP90AA1*	heat shock 90kD protein 1, alpha	NM_001012670	2.190	4.93E-02
Bt.22526.1.S1_at	*HSPB8*	heat shock 22kDa protein 8	NM_001014955	2.110	5.53E-03
Bt.422.1.S1_at	*IGFBP3*	insulin-like growth factor binding protein 3	NM_174556	2.264	3.89E-02
Bt.422.1.S2_at	*IGFBP3*	insulin-like growth factor binding protein 3	NM_174556	2.047	3.47E-02
Bt.29391.1.S1_at	*IL20RA*	interleukin 20 receptor, alpha	XM_585919	2.845	2.95E-02
Bt.24522.3.S1_a_at	*KLF6*	Kruppel-like factor 6	NM_001035271	2.009	4.09E-02
Bt.26572.1.S1_at	*LEPREL1*	leprecan-like 1	NM_001100345	3.043	1.40E-02
Bt.24827.1.A1_at	*LIPG*	lipase, endothelial	XM_586851	-2.721	4.76E-02
Bt.16205.1.A1_at	*LOC511583*	similar to Family with sequence similarity 114, member A1	XM_588946	2.558	3.93E-02
Bt.8586.1.S1_at	*LOC512149 /// LOC512150*	hypothetical LOC512149 /// similar to Myeloid-associated differentiation marker	NM_001104975 /// XM_589599	-2.426	1.40E-02

Bt.9296.1.A1_at	*LOC512486*	interferon-induced guanylate-binding protein 1	XM_590008	2.712	1.74E-02
Bt.25478.1.A1_at	*LOC529947*	similar to signal peptide, CUB domain, EGF-like 2	XM_608409	2.931	2.39E-02
Bt.27140.1.S1_at	*LOC535166*	similar to mKIAA1077 protein	XR_027912	3.574	1.40E-02
Bt.9412.2.S1_at	*LOC781493*	similar to Collagen alpha-1(XIV) chain precursor (Undulin)	XR_027324	2.223	2.00E-03
Bt.20820.1.A1_at	*LTBP1*	latent transforming growth factor beta binding protein 1	NM_001103091	2.862	1.64E-02
Bt.12315.1.S1_at	*LTBP1*	latent transforming growth factor beta binding protein 1	NM_001103091	2.032	3.56E-02
Bt.5011.1.S1_at	*LTBP2*	latent transforming growth factor beta binding protein 2	NM_174385	3.012	1.26E-02
Bt.5011.1.S2_at	*LTBP2*	latent transforming growth factor beta binding protein 2	NM_174385	2.412	3.74E-02
Bt.2560.1.S1_at	*MFAP5*	microfibrillar associated protein 5	NM_174386	3.951	4.15E-02
Bt.13482.2.S1_at	*NOV*	nephroblastoma overexpressed	NM_001102382	2.803	1.33E-02
Bt.27545.1.A1_at	*NRIP3*	nuclear receptor interacting protein 3	NM_001102218	5.603	2.38E-02
Bt.12217.2.S1_at	*NTRK2*	neurotrophic tyrosine kinase, receptor, type 2	NM_001075225	3.066	4.63E-02

Bt.11420.1.A1_at	*P4HA3*	prolyl 4-hydroxylase, alpha polypeptide III	NM_001001598	2.382	3.78E-02
Bt.28162.3.S1_at	*PLN*	phospholamban	NM_001103319	2.253	1.26E-02
Bt.25703.1.A1_at	*PLXDC2*	plexin domain containing 2	NM_001077928	2.204	7.04E-03
Bt.18748.1.A1_at	*PTPRB*	protein tyrosine phosphatase, receptor type, B	XM_582025	2.338	1.44E-02
Bt.24933.1.S1_at	*RARRES1*	retinoic acid receptor responder (tazarotene induced) 1	NM_001075430	3.674	4.48E-02
Bt.10855.1.S1_at	*RGS2*	regulator of G-protein signaling 2, 24kDa	NM_001075596	3.369	2.95E-02
Bt.24212.1.S1_at	*RSPO3*	R-spondin 3 homolog (Xenopus laevis)	NM_001076034	5.454	3.84E-02
Bt.24813.1.A1_at	*RTP4*	receptor (chemosensory) transporter protein 4	NM_001075961	2.349	7.44E-03
Bt.11271.1.S1_at	*SMOC2*	SPARC related modular calcium binding 2	NM_001098134	2.547	4.44E-02
Bt.11061.1.S1_at	*TNC*	tenascin C	NM_001078026	2.304	4.54E-02
Bt.5398.1.S1_at	*TNXB*	tenascin XB	NM_174703	2.168	4.44E-02
Bt.17070.1.S1_at	*TOP1*	topoisomerase (DNA) I	XM_614691	3.217	4.53E-02

Bt.21986.1.A1_a_at	*USP7*	similar to ubiquitin-specific protease 7	XR_042874	2.119	3.76E-02
Bt.26722.1.A1_a_at	*WDFY4*	WDFY family member 4	XM_606192	2.613	1.84E-03
Bt.27254.2.S1_at	*WNT2B*	wingless-type MMTV integration site family, member 2B	NM_001099363	-2.379	1.25E-02
Bt.27254.1.A1_at	*WNT2B*	wingless-type MMTV integration site family, member 2B	NM_001099363	-3.026	4.55E-03
Bt.27155.1.A1_at	*ZNF618*	similar to Zinc finger protein 618	XM_607030	2.093	2.97E-02
Bt.20148.1.S1_at	---			7.586	2.67E-02
Bt.3311.1.S1_at	---			5.194	1.42E-02
Bt.3311.3.S1_at	---			3.711	2.95E-02
Bt.20074.1.S1_a_at	---			3.056	4.25E-02
Bt.15895.1.A1_at	---			2.296	1.70E-02
Bt.18094.1.A1_at	---			2.292	2.19E-03
Bt.21543.1.S1_at	---			2.287	6.93E-03

Bt.22076.1.A1_at	---			2.200	3.66E-02
Bt.26108.1.A1_at	---			2.133	2.04E-02
Bt.21543.2.S1_at	---			2.127	7.01E-03
Bt.13522.1.S1_at	---			2.117	2.83E-02
Bt.17195.1.A1_at	---			2.088	3.84E-02
Bt.22595.1.S1_at	---			-2.840	3.46E-02
